# Morning lighting intervention reduces fatigue and improves sleep in Parkinson disease

**DOI:** 10.1016/j.neurot.2026.e01095

**Published:** 2026-10-21

**Authors:** Jade S. Park, Mengxi Yang, Melba Beltre, Deborah Raymond, Emmanuel During, Adina Wise, Mariel Pullman, Barbara Plitnick, Susan B. Bressman, Mariana G. Figueiro, Rachel Saunders-Pullman

**Affiliations:** a Department of Neurology, Icahn School of Medicine at Mount Sinai and Mount Sinai Beth Israel, 521 W 57th St. Flr 6, New York, NY, 10019, USA; b Light and Health Research Center, Department of Population Health Science and Policy, Icahn School of Medicine at Mount Sinai, 1425 Madison Ave Flr 2, New York, NY, 10029, USA

**Keywords:** Sleep, Light therapy, Circadian rhythm, Fatigue, Parkinson disease

## Abstract

Fatigue and sleep disturbances are common in Parkinson Disease (PD) and significantly reduce quality of life, yet effective pharmacological treatments are limited. Light therapy offers a promising non-pharmacologic approach, but most lighting interventions have focused on improving sleep. Using a novel and easily administered morning lighting intervention, we previously demonstrated improvement in sleep duration. We have now extended the prior study through the addition of 23 individuals to investigate the intervention's effect on fatigue and sleep. In this single-arm intervention study, participants received morning light for 4 consecutive weeks from table or floor luminaires designed to deliver circadian-effective ambient light at home. Objective sleep was measured with wrist-worn actigraphy and subjective sleep, fatigue and mood were assessed using standardized questionnaires. Analysis of pre and post intervention data for the current cohort was performed separately as well as in combination with the 20 participants from the previously reported pilot study. For the 23 current participants, fatigue on the Functional Assessment of Chronic Illness Therapy-Fatigue (FACIT-F) scale improved by 3.9 ± 1.00 points (p = 0.0026). While sleep duration increased by mean 21.3 ± 14.4 min, it was not statistically significant (p = 0.082). When combined with the 20 participants from the prior pilot study, significant improvement in fatigue by 2.8 ± 0.85 points (p = 0.0033) was observed and sleep duration was also significantly increased by 24.7 ± 10.4 min (p = 0.012). These findings support morning light as a novel intervention for fatigue in PD.

## Introduction

Light is a primary regulator of the circadian system, exerting its effects through retinal photoreception and signaling to the suprachiasmatic nucleus (SCN), the central biological clock [1,2]. Through this pathway, light synchronizes physiological rhythms governing sleep, mood, and neuroendocrine function [[3], [4], [5]]. Disruption of circadian rhythm is increasingly recognized as a central contributor to a range of neuropsychiatric and neurodegenerative conditions, providing a promising mechanistic basis for the therapeutic application of light [[6], [7], [8], [9]].

Parkinson disease (PD) is characterized not only by its motor features but also by a substantial burden of non-motor symptoms that markedly reduce quality of life and remain difficult to treat pharmacologically [[10], [11], [12]]. Sleep disruption, fatigue, depression, and cognitive impairment are highly prevalent and often precede the onset of motor symptoms [[12], [13], [14], [15], [16]], suggesting early involvement of circadian dysfunction in disease pathophysiology. Fatigue, which may be a distinct entity from sleepiness, is a particularly challenging symptom to treat [[16], [17], [18], [19], [20]]. As current pharmacologic therapies provide limited relief for non-motor symptoms and may also exacerbate daytime sleepiness and/or fatigue, this further underscores the need for non-pharmacologic approaches [16,19,21,22].

Bright light therapy (BLT) has emerged as a promising intervention targeting circadian disruption in addition to other PD symptoms [[23], [24], [25], [26], [27], [28], [29], [30], [31], [32], [33], [34], [35], [36], [37], [38], [39], [40], [41]]. Early clinical evidence demonstrated that daily exposure to 3000–4000 lux of light at the eye level improved motor and non-motor domains [23]. In another early case series, most patients exposed to BLT exhibited a 30–50% improvement in motor function, particularly with bradykinesia and rigidity. These motor benefits were accompanied by marked improvements in mood, sleep onset and continuity, and overall daily functioning, and light therapy enabled substantial reductions in dopaminergic medication without loss of symptom control [25].

Subsequent controlled and longitudinal studies have reinforced and extended the benefits of BLT [24,26,[28], [29], [30], [31],^34^,[38], [39], [40], [41], [42], [43]]. In a controlled exploratory trial, continued exposure to polychromatic light resulted in significant improvements in Movement Disorder Society-Unified Parkinson's Disease Rating Scale (MDS-UPDRS), quality of life (PDQ-39), sleepiness (Epworth Sleepiness Scale), depression, and anxiety, with consistent gains observed over short-term follow-up [30]. Long-term data further from the same group highlight the durability and scope of these effects. In a 10-year retrospective study, circadian-targeted light therapy produced early and sustained improvements in sleep, fatigue, and depression, with benefits emerging within the first month and persisting over years in adherent patients [38].

Recent findings have highlighted the variability in non-motor treatment response to BLT in PD [[33], [34], [35], [36], [37], [38], [39], [40], [41], [42]]. A large randomized controlled trial reported that evening light exposure, alone or combined with morning light did not meet primary sleep endpoints [39], underscoring the importance of treatment parameters such as timing, light level, delivery method, and adherence. Notably, in this study, fatigue improved as a secondary outcome, particularly in participants receiving morning light [39]. Ideal light timing and delivery method may be important for effectively promoting circadian entrainment in PD [24].

To further assess the effect of light treatment on neurodegenerative disease, we previously developed a novel, tailored lighting intervention designed to deliver ambient circadian-effective light via floor or tabletop luminaires [37]. This approach specifically targets the photoreceptors involved in circadian phototransduction. The circadian system is most sensitive to short-wavelength (blue) light and requires higher light levels than the visual system for activation. This intervention, when delivered in the morning (within 2 h of waking), has demonstrated efficacy in improving sleep, mood, and behavior in individuals with Alzheimer's disease and related dementias (ADRD) [44]. In our pilot study in PD, the same tailored morning lighting intervention resulted in significant improvements in sleep duration, with reduction in fatigue that did not reach statistical significance [37].

The present study aims to further evaluate the effects of this tailored morning lighting intervention on fatigue and sleep in individuals with PD. Specifically, we assess outcomes in an expanded cohort of 23 additional participants and report results for both the new cohort and the combined sample. By focusing on fatigue as a main outcome alongside sleep, this study seeks to clarify the therapeutic potential of circadian-targeted light exposure in addressing key non-motor symptoms of PD.

## Materials and methods

From January 2023 through May 2024, 23 additional participants were recruited from the Bonnie and Tom Strauss Movement Disorders Center at Mount Sinai in New York, NY including five cross-enrolled from the NIH NINDS/NIA U01-NS107016 study (registered as NCT05757414). Ethical approval for the study was received from the Icahn School of Medicine at Mount Sinai Institutional Review Board. The study was conducted in accordance with the Declaration of Helsinki, and informed consent was obtained for all participants. In order to be included in the study, participants were not required to have sleep complaints.

The study design was as previously reported for our pilot study [37]. Study duration spanned five weeks: baseline data collection during week 1, lighting intervention for weeks 2–5, and a concluding second round of data collection during week 5, which was the last week of the intervention [37]. The 4-week intervention period was selected based on our previous work with AD showing that the benefits of light are maintained, but no longer increase after 3 weeks of light exposure. This duration was also planned to reduce participant burden with the study protocol.

The lighting intervention (Fig. 1) was developed by the Light and Health Research Center at the Icahn School of Medicine at Mount Sinai [37]. The floor and tabletop luminaires combined light spectrum and light levels designed to maximally stimulate the human circadian system based on the mathematical model of circadian phototransduction proposed by Rea and colleagues [45,46]. According to the model, circadian stimulus (CS) is calculated from circadian-effective light (CL_A_), which is a biophysical construct relating the spectral sensitivity of the human retina to optical radiation for stimulation of the master biological clock in the SCN. Above the modeled threshold (CS = 0.1) of the circadian system, CL_A_ values are transformed into CS values until they reach a point of saturation (CS = 0.7), beyond which greater CL_A_ values do not incrementally affect CS. Previous research among people with ADRD has shown that CS > 0.3 during the day (approximately >350–400 photopic lux at the eye level) and <0.1 (<50 photopic lux at the eye level) during the evening improved sleep, mood, and behavior in that population [44,47]. This CS value is approximately six times lower than what is delivered by a traditional light box, potentially making it more comfortable for users, especially older adults who may be more sensitive to glare. The luminaires were installed in a space where each participant reported spending most of their morning. Participants were asked to remain in that space for at least 2 h after waking, but to start no later than 9 a.m. The timing was selected because morning light is known to promote circadian entrainment in humans. Although doses of morning light therapy shorter than 2 h has been shown to be sufficient for improving sleep [24], the 2 h dose was used to account for breaks and to ensure participants had a net 1 h exposure time. Moreover, to ensure the prescribed light level at the eye, participants were instructed to maintain the installed placement of their assigned luminaires at 40–50 cm from their eyes. Evening light in each participant's home was neither prescribed nor measured for this study.

**Fig. 1:**
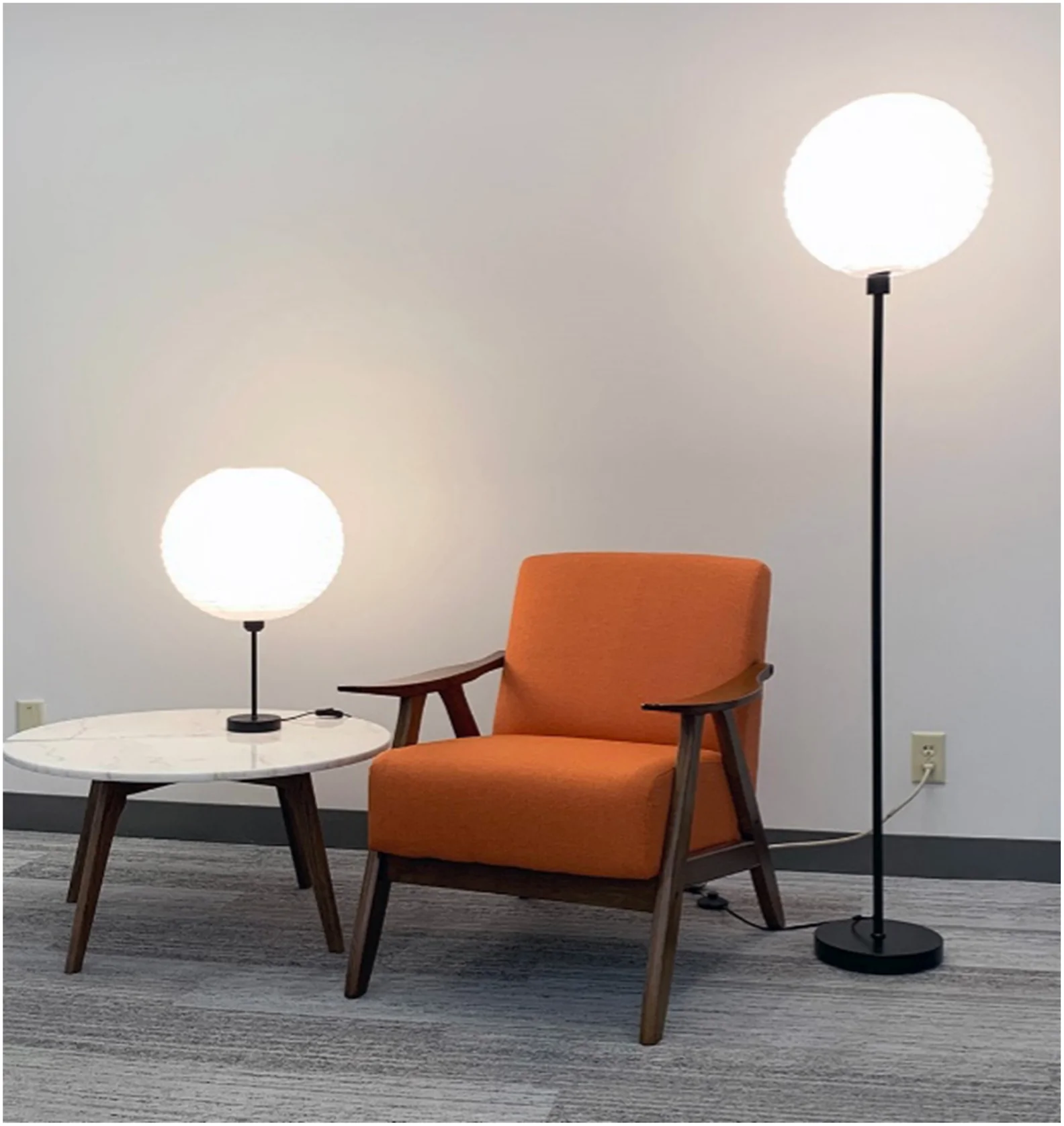
Floor and table luminaires used in our study.

Participants were offered the choice of a tabletop or floor luminaire, both fitted with a spherical paper shade (40 cm diameter) to softly diffuse the light and thereby reduce glare (Fig. 1). When installed at each participant's residence, the height of the tabletop luminaire was 58 cm above the table's work surface; the height of the floor luminaire was 170 cm. The luminaire was fitted with a light emitting diode (LED) light source. The light source (model # OM200/830/LED, Feit Electric, Pico Rivera, CA, USA) emitted 3050 lumens at a correlated color temperature (CCT) of 3000 K (neutral-to-warm white in color). The lighting characteristics of the light source was measured in our lab via photometer (model X91, Gigahertz-Optik, Türkenfeld DE) and is summarized in Supplementary Table 1. The only difference between the current and pilot study protocol was that the previous pilot allowed participants to choose Re-Timer glasses or luminaires. However, only three participants chose the glasses in the previous pilot. Thus, these were not offered in the current study, where tabletop or floor luminaires were the only options.

Objective sleep measures were recorded with wrist-worn actigraphy at baseline and in the final week of the study for pre-and-post intervention comparisons. Each participant wore an actigraph (Philips Respironics Actiwatch-L, Murraysville, PA, US) for seven days on their non-dominant wrist, awake and asleep, during data collection periods. Using the actigraphy data and the manufacturer software, sleep duration, sleep onset latency, wake after sleep onset, and sleep efficiency were determined (Table 2). During the assessment weeks, each participant wore a Daysimeter [48], a calibrated personal light measuring device (Light and Health Research Center, Troy, NY, US), as a pendant around their neck to record individual light exposure data which allowed adherence to the lighting intervention to be verified.

**Table 1:** Baseline demographics and clinical features of current and combined cohorts.

Demographic variable	Current cohort n = 23	Combined cohort^a^ n = 43
**Age,** yrs, mean ± SD (range)	69.5 ± 12.7 (26.0, 81.2)	70.7 ± 11.3 (26.0, 85.4)
**Age at onset,** yrs, mean ± SD (range)	59.5 ± 11.9 (25.0, 74.0)	61.1 ± 12.1 (25.0, 81.0)
**Sex**		
Women	7 (30%)	19 (44%)
Men	16 (70%)	24 (56%)
**Disease duration,** yrs, mean ± SD (range)	10.3 ± 6.0 (0.7, 27.0)	9.6 ± 5.6 (0.7, 27.0)
**MoCA score,** baseline	23.6 ± 4.2 (13.0, 30.0)	24.6 ± 3.9 (13.0, 30.0)
**LEDD (mg)**, mean ± SD (range)	639.1 ± 502.1 (0.0, 1882.5)	614.4 ± 434.1 (0.0, 1882.5)
**H&Y stage**^b^		
1	6 (27%)	8 (20%)
2	10 (45%)	21 (53%)
3	5 (23%)	8 (20%)
4	1 (4.5%)	3 (7.5%)

a Combined cohort includes those in current study as well previously reported pilot cohort using same methodology (Yoo et al., 2024).

b H&Y staging only available for n = 22 in current cohort*. Abbreviations: H&Y,* Hoehn & Yahr scale; *LEDD,* Levodopa Equivalent Daily Dosage; *MoCA,* Montreal Cognitive Assessment.

**Table 2:** Univariate comparisons of clinical outcomes of current and combined cohorts.

	Current cohort (n = 23)				Combined cohort (n = 43)			
	Baseline	Post- intervention	Change (SE)	p-value	Baseline	Post- intervention	Change (SE)	p-value
*Actigraphy*								
Sleep duration (min)	442.2 (87.9)	465.2 (82.8)	21.26 (14.4)	0.082	432.6 (84.9)	458.3 (89.8)	24.72 (10.40)	0.012∗
Sleep onset latency	10.2 (12.2)	12.9 (22.0)	3.10 (5.17)	0.72	12.3 (12.1)	14.6 (19.3)	2.48 (3.39)	0.76
WASO	70.0 (32.2)	75.8 (43.2)	7.75 (8.62)	0.81	56.4 (29.9)	63.8 (41.6)	8.15 (5.56)	0.92
Sleep efficiency (%)	80.3 (7.8)	79.6 (12.2)	−1.26 (2.26)	0.70	82.4 (9.5)	81.9 (10.5)	−0.76 (1.45)	0.70
*Questionnaires*								
Fatigue (FACIT-F)	29.2 (11.1)	32.0 (9.5)	3.94 (1.00)	0.0026∗	31.7 (12.2)	34.3 (10.5)	2.77 (0.85)	0.0033∗
Sleep (PDSS-2)	19.3 (8.0)	18.8 (6.8)	−0.06 (1.61)	0.49	18.0 (7.6)	17.2 (6.6)	−0.51 (0.95)	0.32
Daytime sleepiness (ESS)	9.4 (5.4)	8.6 (3.7)	−0.24 (0.77)	0.40	7.8 (5.4)	7.6 (4.2)	0.00 (0.44)	0.50
Depression (HAM-D)	10.5 (4.9)	8.9 (5.1)	−1.65 (0.79)	0.045∗	10.9 (5.7)	9.6 (5.8)	−1.14 (0.55)	0.034∗
Anxiety (HAM-A)	11.4 (5.1)	9.7 (6.3)	−0.88 (1.11)	0.25	11.1 (5.7)	9.7 (5.8)	−0.97 (0.71)	0.12
Apathy (AS)	13.4 (8.0)	15.1 (10.8)	1.88 (0.97)	0.94	12.5 (7.6)	13.2 (9.3)	0.76 (0.74)	0.82

Mean (SD). Results for univariate paired 1-sided *t*-test shown. *Abbreviations: AS,* Apathy Scale; *ESS,* Epworth Sleepiness Scale; *FACIT-F,* Functional Assessment of Chronic Illness Therapy-Fatigue Scale; *HAM-A,* Hamilton Anxiety Rating Scale; *HAM-D,* Hamilton Depression Rating Scale; *PDSS-2,* Parkinson's Disease Sleep Scale 2; *WASO*, Wake After Sleep Onset. *Clinical cutoff scores: AS*(>14) [49], *ESS*(>10) [50], *FACIT-F*(<30) [51], *HAM-A*(≥8) [52], *HAM-D* (≥8) [53], *PDSS-2*(≥15) [54].

Subjective measures were collected pre-and-post-lighting intervention (weeks 1 and 5) using questionnaires, which were completed by participants or by participants with care partner input, assessing fatigue with the Functional Assessment of Chronic Illness Therapy-Fatigue (FACIT-F) scale [55], subjective sleep with the Parkinson's Disease Sleep Scale 2 (PDSS-2) [56]*,* and sleepiness with the Epworth Sleepiness Scale (ESS) [50]*.* Psychiatric features were assessed using the Hamilton Depression Rating Scale (HAM-D) [57]*,* Hamilton Anxiety Rating Scale (HAM-A) [58], and Apathy Scale [49] (Table 2).

As prior benefits with lighting had been demonstrated, pre-and-post-lighting intervention outcomes were compared with one-tailed paired t-tests for normally distributed data and paired sample Wilcoxon signed rank test for non-parametric data [37]. Outcomes included objective variables (sleep duration, sleep time, onset latency, wake after sleep onset [WASO], sleep efficiency, and percent sleep) and subjective sleep measures (FACIT-F, ESS, PDSS-2 as well as HAM-A, HAM-D and AS). Regression models were performed for those outcomes demonstrating statistical significance in the univariate analyses, and included sleep duration, FACIT-F and HAM-D. These were evaluated for both the current and combined cohorts and included adjustment for independent variables of age, sex, and disease duration. Statistical analysis was run using Stata 17 statistical software (StatCorp, College Station, TX, US) and open-source statistical programming language R and integrated development environment RStudio (The R Foundation for Statistical Computing, Vienna, Austria). The statistical significance threshold was p < 0.05. If a question was incomplete or missing in a questionnaire, the questionnaire was excluded. We performed analyses first in the current cohort alone (n = 23) and, second, in the combined cohort including the pilot cohort (n = 43).

## Results

Mean age for the 23 participants in the current cohort was 69.5 ± 12.7 years, mean disease duration was 10.3 ± 6.0 years and 30% were female, with further clinical characteristics in Table 1. In addition, 13% had *Leucine-rich repeat kinase 2* (*LRRK2*) G2019S variants and 17% harbored variants in *Glucosylceramidase beta 1* (*GBA1*) gene. Ten screened positive on the Mayo Sleep Questionnaire item 1 supportive of REM sleep behavior disorder [59], although formal sleep study was not performed. Clinical features from the pilot and extension cohort are shown in Table 1. Overall, the cohort characteristics were similar in both groups, however, there were fewer *LRRK2* PD in the current cohort compared with the pilot respectively (13% vs. 40%).

Changes in sleep measures and questionnaire data are shown in Table 2. In the current group alone, mean sleep duration from pre-to-post intervention increased by 21.3 ± 14.4 min (p = 0.082) (Fig. 2). Fatigue improved as shown by an increase of 3.9 ± 1.00 points in the FACIT-F scale (p = 0.0026) (Fig. 3), and depression improved as shown by a decrease of 1.7 ± 0.79 points in the HAM-D scale (p = 0.045). No other outcomes were significant. In regression models adjusting for age, sex, and duration of PD (Table 3), the effect of the lighting intervention remained significant only for fatigue in the current cohort (β = 3.8, 95% CI: 1.5 to 6.2, p = 0.001). Participant age was negatively associated with HAM-D change (β = −0.17, 95% CI: -0.33 to −0.01, p = 0.04).

**Fig. 2:**
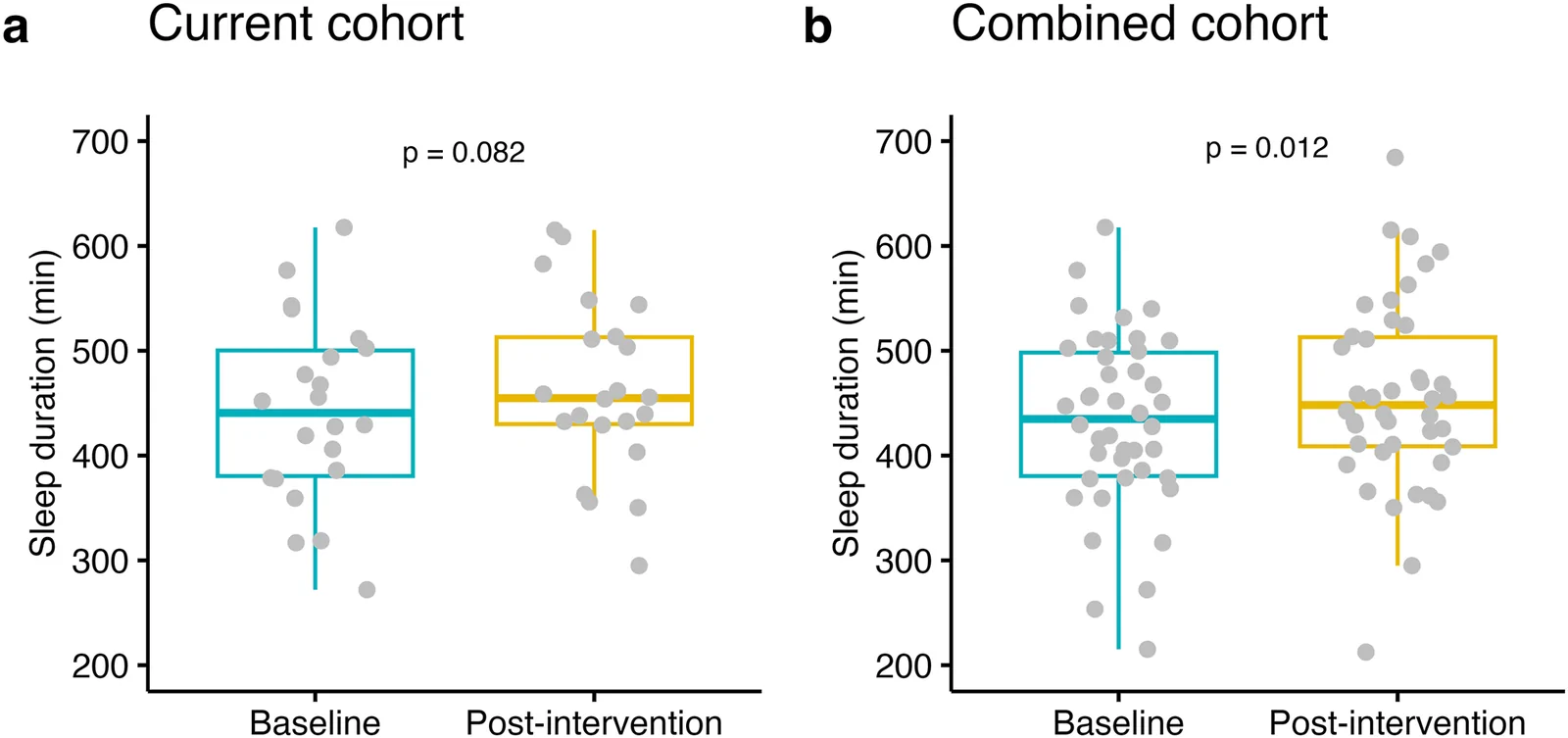
**Sleep duration in pre and post lighting intervention** for 23 participants in **(a)** the current cohort, and **(b)** 43 participants in the combined cohort. The horizontal line in each box-and-whisker plot represents the median. Mean sleep duration was not significantly greater post-intervention compared to baseline for the current cohort, however, increased significantly in the combined cohort.

**Fig. 3:**
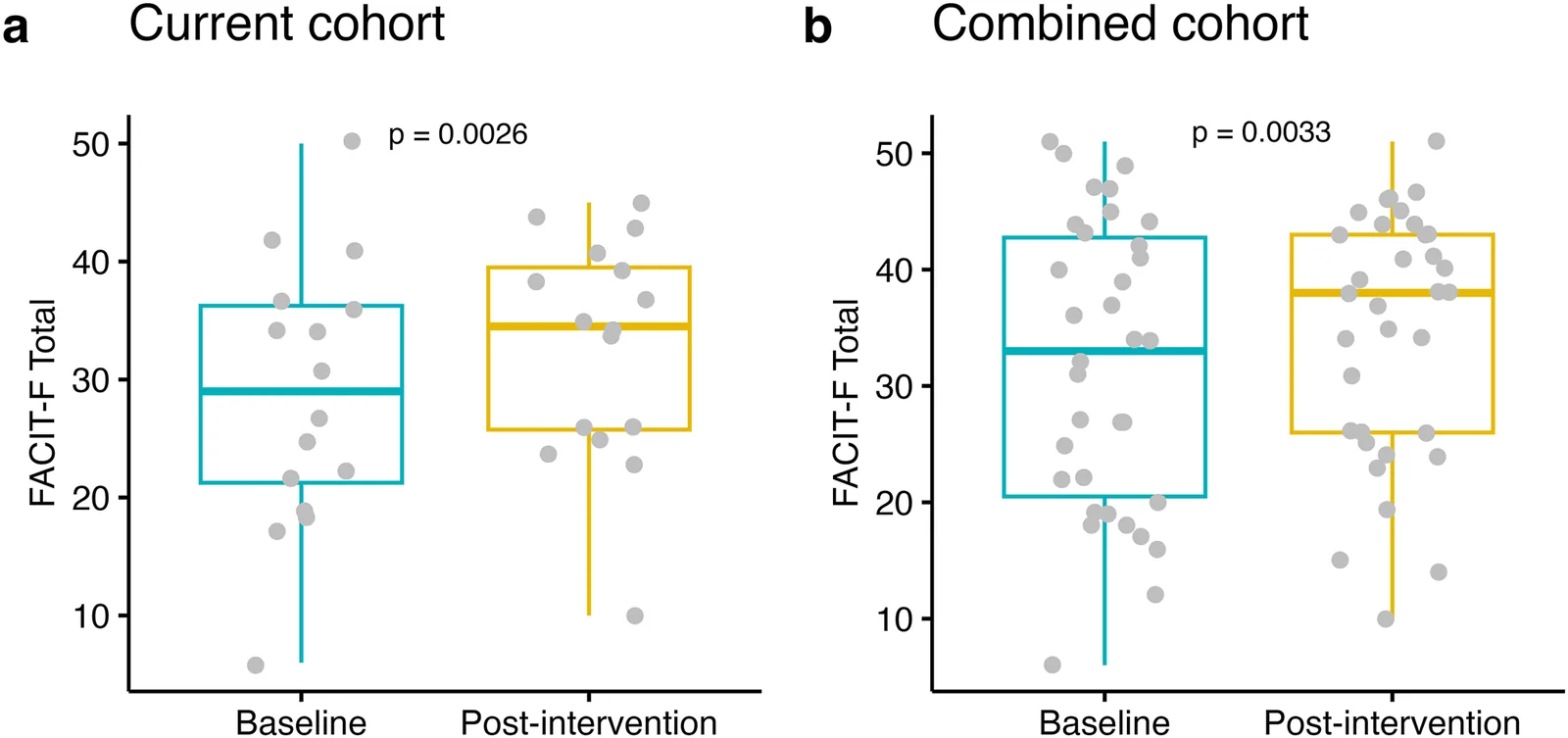
**Fatigue pre- and post-lighting intervention** for **(a)** 23 participants in the current cohort, and **(b)** 43 participants in the combined cohort. The horizontal line in each box-and-whisker plot represents the median. Mean FACIT-F was significantly greater post-intervention compared to baseline for the current and combined cohorts. *FACIT-F,* Functional Assessment of Chronic Illness Therapy-Fatigue Scale.

**Table 3:** Regression estimates modeling the effect of lighting intervention on fatigue, sleep duration and depression in current and combined cohorts.^a^

**A.** Current cohort	Model A1. Fatigue (FACIT-F total score)			Model A2. Sleep duration (min)			Model A3. Depression (HAM-D total score)		
	β	95% CI	p-value	β	95% CI	p-value	β	95% CI	p-value
**Intervention effect**	3.8	1.5, 6.2	0.001∗	21	−7.3, 50	0.14	−1.7	−3.5, 0.04	0.055
**Age**	0.29	−0.07, 0.65	0.12	0.80	−2.2, 3.8	0.6	−0.17	−0.33, −0.01	0.040∗
**Disease duration (years)**	0.07	−0.76, 0.90	0.9	−3.4	−10, 3.5	0.3	0.17	−0.21, 0.54	0.4
**Female sex**	3.3	−6.5, 13	0.5	0.31	−80, 80	>0.9	−1.3	−5.7, 3.1	0.6

**B.** Combined cohort	Model B1. Fatigue (FACIT-F total score)			Model B2. Sleep duration (min)			Model B3. Depression (HAM-D total score)		

β	95% CI	p-value	β	95% CI	p-value	β	95% CI	p-value
**Intervention effect**	2.7	0.86, 4.6	0.004∗	25	4.2, 45	0.018∗	−1.2	−2.4, −0.02	0.047∗
**Age**	0.23	−0.05, 0.51	0.11	1.6	−0.59, 3.7	0.2	−0.11	−0.26, 0.04	0.2
**Disease duration (years)**	0.19	−0.45, 0.83	0.6	−4.6	−9.4, 0.30	0.066	0.12	−0.22, 0.46	0.5
**Female sex**	6.9	−0.20, 14	0.057	−13	−67, 41	0.6	0.86	−2.9, 4.6	0.7

a Regression estimates modeling the effect of the lighting intervention effect on fatigue (*FACIT-F*, Functional Assessment of Chronic Illness Therapy-Fatigue Scale total score, Model A1 and B1), sleep duration (in minutes, Models A2 and B2) and depression, (*HAM-D*, Hamilton Depression Rating Scale, Model A3 and B3) in current and combined cohorts adjusting for participant age, duration of disease, and sex.

In the combined cohort, mean sleep duration from pre-to-post intervention increased by 24.7 ± 10.4 min (p = 0.012) (Table 2). Fatigue improved as shown by an increase in 2.8 ± 0.85 points in the FACIT-F scale (p = 0.0033) (Fig. 3) and depression improved as shown by a decrease of 1.1 ± 0.55 points in the HAM-D scale (p = 0.034). These outcomes remained significant in 2-tailed tests with the exception of depression. In regression models adjusting for age, sex, and duration of PD (Table 3), the effect of the lighting intervention on FACIT-F remained significant, improving by on average 2.7 points (95% CI: 0.86 to 4.6, p = 0.004) from pre-to-post-intervention. Similarly, sleep duration increased by an average of 25 min (95% CI: 4.2 to 45, p = 0.018) following circadian effective lighting intervention in the adjusted model. Depression improved by 1.2 points (95% CI: -2.4 to −0.02, p = 0.047). Age, female sex, and PD duration terms were not significant in the adjusted models. Sensitivity analyses were conducted excluding the three participants from the pilot study who used Re-Timer glasses. Effect sizes were similar with regard to improvement in sleep duration, fatigue (FACIT-F), and depression (HAM-D) (Supplementary Table 2A and 2B).

To verify compliance, we evaluated the Daysimetry data for the CS exposure between 6 a.m. and noon and between 7 a.m. and noon. In both cases, the CS exposure during the last week of the intervention was significantly greater than before the intervention. (For 6–12 p.m., baseline CS 0.25±0.11 and Intervention 0.3±0.12, *t*-test p = 0.0001 and for 7–11 a.m. baseline CS 0.28±0.09 and intervention 0.33± 0.10, p = 0.0003). More importantly, we showed that the average CS values during the intervention reached the average target CS value of 0.3.

## Discussion

Our study highlights the promise of this noninvasive lighting intervention to address challenging non-motor symptoms in PD by demonstrating improvements in fatigue and sleep duration in the combined cohort. The findings are particularly notable for fatigue, a common feature of PD with limited pharmacologic treatment options despite its substantial impact on quality of life [16,19]. Moreover, the lighting intervention was easy to administer, well tolerated, and associated with minimal participant burden. Although our study was modest in size, the findings should be interpreted within the broader context of prior studies examining circadian and sleep disturbances and light-based interventions in PD, many of which support beneficial effects of supplemental lighting on fatigue, sleep, and mood [[23], [24], [25],[28], [29], [30], [31], [32],^34^,[37], [38], [39], [40], [41]].

Circadian disruption is common and multifactorial in PD [7,60,61]. Light is a primary regulator of circadian rhythmicity [62,63], and both insufficient daytime light exposure and excessive nighttime light exposure may contribute to circadian disruption. Individuals with PD have been shown to receive lower levels and shorter durations of daytime light exposure, as well as greater nighttime light exposure, than healthy controls [64]. Increased daytime light exposure has been associated with reduced circadian disruption and related sequelae across several clinical conditions. Light therapy is an established treatment for seasonal affective disorder, improving mood during periods of reduced winter daylight availability [65,66]. Light therapy delivered through light boxes has also been associated with reduced fatigue in cancer patients [67,68] and improved sleep in individuals with AD [69] and PD [25,26,30,38].

Light therapy has been investigated in PD for both motor and non-motor symptoms, including sleep onset and continuity [25], daytime sleepiness and sleep quality [28], and depression [[23], [24], [25],^32^,^38^]. However, studies have varied considerably in treatment duration, light levels, timing, frequency, and participant burden, contributing to mixed findings. Although subjective improvements have generally been positive, evidence for objective actigraphic sleep improvement has been inconsistent [28,31,37]. Variability in dose, delivery method, and adherence likely contributed to this heterogeneity. We and others have shown that morning light exposure, specifically designed to target the circadian system, can improve sleep in PD [24,33,37]. The present study further demonstrated benefits in objectively measured sleep, fatigue, and mood, all of which are common non-motor symptoms in PD. Sleep and circadian disruption in PD may also impair glymphatic function and alpha-synuclein clearance [70,71], potentially accelerating neurodegeneration [72,73].

Fatigue has emerged as one potential positive outcome in light therapy studies in PD [38,39]. It is of interest that fatigue and depression improved, but not apathy. While both may occur in PD, fatigue is multidimensional. Features associated with fatigue may overlap with apathy, however these two entities may also be separate. In particular, there has been focus particularly on delineating core components of apathy that differ from fatigue, as well as depression and anxiety [74]. The pathophysiology of fatigue in PD remains poorly understood but may involve frontostriatal and limbic circuit dysfunction, serotonergic, glutamatergic, and dopaminergic pathways, and, more recently, immune-related mechanisms, as reviewed by Bojitos et al. [16]. Notably, the current cohort demonstrated significant improvement in fatigue despite limited improvement in sleep, raising the possibility that lighting interventions may exert effects beyond circadian modulation alone.

Our findings differ from some prior studies in demonstrating improvements in objective sleep measures [31,39]. Differences across studies may reflect methodological variation and highlight the importance of intervention characteristics, such as timing, duration, and delivery methods that influence circadian responses [75]. The observed improvements in both fatigue and sleep with morning light exposure suggest that intervention timing might be a determinant of treatment effectiveness.

An important consideration in morning light therapy is that participants were not studied in a controlled lighting environment, such as nursing homes, and therefore received light exposure outside the intervention itself. On average, morning light exposure between 6:00 a.m. and noon produced a circadian stimulus (CS) of 0.25 before intervention and 0.30 after intervention. Although this modest increase supports adherence, the relatively small change may partially explain the substantial variability observed in the data. In addition to timing, heterogeneity in response to light suggests that individual factors, including genotype, sex, and baseline sleep and circadian disturbances, may influence treatment efficacy [76]. Delivery method and participant adherence may also affect efficacy of the treatment response.

Our findings also further support the possibility that light therapy may serve as an adjunctive treatment for depression in PD [31,77]. Circadian-effective lighting like that used in our study has reduced development of depression in hospitalized myeloma patients undergoing autologous stem cell transplantation and improved mood in individuals with ADRD [44,78]. Although pharmacologic therapies for depression in PD are more widely available than those for fatigue, nonpharmacologic and noninvasive therapies remain important adjunctive treatment options [79].

Strengths of our study include the demonstration of improvements in sleep using a noninvasive, nonpharmacologic, and well-tolerated, low glare intervention associated with minimal participant burden compared with prior light therapy studies. We improved the accuracy of sleep measurement by using actigraphy with a dedicated bedtime marker and reduced participant burden by eliminating the need for sleep diaries [39,64] and manual light therapy logs [39]. Adherence to the lighting intervention was objectively assessed with the Daysimeter, which enabled monitoring of both intervention exposure and exposure to external light such as daylight.

While our pre-post, within-subject study design has the advantage of allowing participants to remain on the same level of medications and serve as their own control, a placebo effect can always be a factor. We do not believe our specific results are solely due to placebo effects, however. The effect size, as measured by Cohen's f^2^ for the regression for the combined and current analyses were medium to large for the FACIT-F scale (0.242 and 0.665 respectively) (Supplementary Table 3). This is consistent with another lighting study in PD that evaluated subjective sleep measures, and showed small Cohen's effect size of the placebo [43]*.* Further, previous work evaluating the impact of light therapy on cancer-related fatigue using the same fatigue scale showed that while there is a potential placebo effect in the first two weeks of treatment, this effect does not exist after 4 weeks [68], which was the length of our study.

Similar effects were shown in a lighting intervention trial using other subjective scales. For example, in a crossover, placebo-controlled lighting intervention trial for AD, we showed that the effect size (Cohen's d) for the Cornell Scale for Depression in Dementia was 0.58 for the active intervention and 0.19 for the placebo intervention. Further, placebo effects are more likely to be present with subjective measures, such as the subjective rating scales for daytime sleepiness, fatigue, apathy and depression. They are less likely to have an effect on actigraphy measures of recorded sleep as this is an objective measure. Notably, the effect size for sleep duration was small, but as sleep duration significantly affects quality of life, even incremental changes can be meaningful, and double-blind studies will need to be powered for this small effect size if sleep duration is a primary outcome. In fact, the present results are significant because they can serve as the basis for powering randomized, double-blind placebo-controlled trials with morning light therapy for PD patients.

Our study was limited in that it focused exclusively on non-motor symptoms related to sleep and did not evaluate motor outcomes as others have done [[24], [25], [26],^28^,^30^,^38^]. Fatigue assessment represented another potential limitation. Although the FACIT-F scale has demonstrated good reliability and comparability across PD and non-PD populations [80,81], future studies could incorporate additional measures such as the Multidimensional Fatigue Inventory [82], Fatigue Severity Scale [83], or the Movement Disorder Society Non-Motor Symptoms Questionnaire [84]. Generalizability may also be limited because the study originally emphasized *LRRK2* and *GBA1* variant carriers. Of note, we did not restrict enrollment to participants with baseline sleep complaints, which may better reflect the broader PD population but could have also reduced the ability to detect benefit among those most likely to show change with the intervention.

We also did not exclude participants receiving melatonin therapy for REM sleep behavior disorder or insomnia, as discontinuation could have worsened underlying sleep symptoms and increased participant burden. The inclusion of participants using melatonin may represent an additional limitation because melatonin acts as a circadian phase modulator and has been studied in combination with BLT in PD. However, sensitivity analyses excluding melatonin users continued to show improvements in sleep duration and fatigue remained significant after excluding melatonin users (Supplementary Table 4).

Clinically, our findings support the potential of morning circadian-effective light therapy as a well-tolerated, low burden, nonpharmacologic intervention for challenging non-motor symptoms in PD. Longer-duration studies, including crossover and randomized controlled trials of morning circadian-effective lighting, are warranted to confirm durability of benefit and refine personalized treatment approaches for fatigue, sleep disturbance, and depression in genetically diverse PD populations. Thus, this lighting approach may represent a practical adjunct to standard care given the current limitations of pharmacologic therapies targeting sleep disruption and fatigue, and further sets the stage for placebo controlled trials.

## Disclosure statement

All authors report no disclosures relevant to the manuscript that are separate from the study funding above.

## Author contributions

Jade S. Park: Writing-original draft, Investigation, Conceptualization, Writing-review & editing.

Mengxi Yang: Writing-review & editing, Investigation, Data Curation.

Melba Beltre: Writing-review & editing, Investigation, Data Curation.

Deborah Raymond: Writing-review & editing, Methodology, Investigation.

Emmanuel During: Writing-review & editing, Conceptualization.

Adina Wise: Writing-review & editing, Investigation, Data Curation.

Mariel Pullman: Writing-review & editing, Investigation, Data Curation.

Barbara Plitnick: Writing-review & editing, Methodology, Investigation, Data Curation.

Susan B. Bressman: Writing-review & editing, Conceptualization.

Mariana G. Figueiro: Writing-original draft, Methodology, Investigation, Conceptualization,

Data Curation, Writing-review & editing, Funding Acquisition, Supervision.

Rachel Saunders-Pullman: Writing-original draft, Methodology, Investigation,

Conceptualization, Data Curation, Writing-review & editing, Funding Acquisition, Supervision.

## Funding

NIH-NINDS 1U01NS107016-01A1 (RSP), NIH-NINDS/NIA 1U01NS107016–03S1 (RSP, DR, MY), Silverstein Foundation for Parkinson's with GBA (JSP), Empire Clinical Research Investigator Program (MP, MB, AW), NIH-NIA 2R01AG034157 (MF, BP), Bonnie and Tom Strauss Chair in Neurology (RSP).

## Declaration of competing interest

The authors declare the following financial interests/personal relationships which may be considered as potential competing interests:

Jade S Park reports financial support was provided by the Silverstein Foundation for Parkinson's with GBA. Rachel Saunders-Pullman reports that financial support was provided by the Bachmann-Strauss Professorship, the National Institute of Neurological Disorders and Stroke, the National Institute on Aging. Mengxi Yang reports financial support was provided by National Institute of Neurological Disorders and Stroke, Empire Clinical Research Investigator Program, the Michael J Fox Foundation for Parkinson's Research, and the Bigglesworth Foundation. Deborah Raymond reports that support was provided by the National Institute of Neurological Disorders and Stroke. Mariel Pullman, Melba Beltre and Adina Wise report that financial support was provided by the Empire Clinical Research Investigator Program. Mariana G. Figueiro reports the financial support was provided by the National Institute on Aging. Barbara Plitnick reports the financial support was provided by the National Institute on Aging. If there are other authors, they declare that they have no known competing financial interests or personal relationships that could have appeared to influence the work reported in this paper.
