# Supplementary material for: Morning lighting intervention reduces fatigue and improves sleep in Parkinson disease
Source: Neurotherapeutics. 2026 Oct 21;24(1):e01095. doi: 10.1016/j.neurot.2026.e01095 (PMC13717183; doi:10.1016/j.neurot.2026.e01095)
Supplement: Supplementary file 1 [file mmc1.docx]

**Supplementary Table 1**:

**Mean radiometric and colorimetric values for the light conditions**

| Lighting Intervention Characteristics |  |
| --- | --- |
| Vertical photopic illuminance (lx) | 363.10 |
| Vertical irradiance (W·m-2) | 1.09 |
| Correlated color temperature (K), nominal | 3031 |
| Color rendering index | 83.60 |
| CLA | 274 |
| CS | 0.30 |
| Melanopic equivalent daylight illuminance | 167 |
| CIE S-cone irradiance (W·m-2) | 0.12 |
| CIE M-cone irradiance (W·m-2) | 0.43 |
| CIE L-cone irradiance (W·m-2) | 0.5973 |
| CIE rhodopic irradiance (W·m-2) | 0.29 |
| CIE melanopic irradiance (W·m-2) | 0.22 |
| Note: Value were calculated via the publicly available online CS Calculator 2.0 at [https://cscalc.light](https://cscalc.light/) health.org | |

**Supplementary Table 2:**

| **2A. Sensitivity analysis excluding Re-Timer glasses users (n=3)** | | | |
| --- | --- | --- | --- |
|  | **Combined cohort (n = 40)** | | |
|  | Baseline | Post-intervention | p-value |
| Actigraphy |  |  |  |
| **Sleep duration (min)** | 430.0 (86.6) | 455.4 (91.6) | **0.02** |
| **Sleep onset latency** | 12.6 (12.5) | 14.8 (19.9) | 0.75 |
| **WASO** | 58.9 (29.4) | 66.9 (41.5) | 0.92 |
| **Sleep efficiency (%)** | 81.6 (9.4) | 81.1 (10.3) | 0.71 |
| Questionnaires |  |  |  |
| **Fatigue (FACIT-F)** | 30.8 (12.3) | 33.5 (10.5) | **<0.001** |
| **Sleep (PDSS-2)** | 18.6 (7.6) | 17.9 (6.4) | 0.36 |
| **Daytime sleepiness (ESS)** | 8.0 (5.5) | 7.7 (4.4) | 0.50 |
| **Depression (HDRS)** | 11.4 (5.6) | 9.8 (5.9) | **0.01** |
| **Anxiety (HAM-A)** | 11.5 (5.7) | 9.9 (6.0) | 0.11 |
| **Apathy (AS)** | 12.7 (7.9) | 13.3 (9.5) | 0.80 |
| Mean (SD). Results for univariate paired 1-sided t-test shown. *Abbreviations: AS,* Apathy Scale; *ESS,* Epworth Sleepiness Scale; *FACIT-F,* Functional Assessment of Chronic Illness Therapy-Fatigue Scale; *HAM-A,* Hamilton Anxiety Rating Scale; *HAM-D,* Hamilton Depression Rating Scale; *PDSS-2,* Parkinson’s Disease Sleep Scale 2; *WASO*, Wake After Sleep Onset. *Clinical cutoff scores: AS*(>14) [54], *ESS* (>10) [51], *FACIT-F*(<30) [S1], *HAM-A*(≥8) [S2], *HAM-D* (≥8) [S3], *PDSS-2*(≥15) [S4] | | | |

| **2B. Sensitivity regression analyses excluding Re-Timer glasses users (n=3)** | | | | | | | | | |
| --- | --- | --- | --- | --- | --- | --- | --- | --- | --- |
|  | **FACIT-F** | | | **Sleep duration** | | | **HDRS** | | |
|  | Beta | 95% CI | p-value | Beta | 95% CI | p-value | Beta | 95% CI | p-value |
| **Time** | 2.8 | 0.87, 4.8 | **0.005** | 24 | 2.1, 46 | **0.032** | -1.4 | -2.6, -0.27 | **0.015** |
| **Age** | 0.27 | -0.02, 0.56 | 0.068 | 1.7 | -0.51, 4.0 | 0.13 | -0.13 | -0.29, 0.02 | 0.10 |
| **Disease duration (years)** | 0.19 | -0.46, 0.83 | 0.6 | -5.3 | -10, -0.35 | **0.036** | 0.12 | -0.23, 0.47 | 0.5 |
| **Female sex** | 7.3 | 0.26, 14 | **0.042** | -10 | -65, 45 | 0.7 | 0.79 | -3.0, 4.6 | 0.7 |
| Regression estimates modeling the effect of the lighting intervention effect on fatigue (*FACIT-F*, Functional Assessment of Chronic Illness Therapy-Fatigue Scale total score, Model A1 and B1), sleep duration, and depression, (*HAM-D*, Hamilton Depression Rating Scale) in the combined cohort excluding Re-Timer glasses users, adjusting for participant age, duration of disease, and sex. | | | | | | | | | |

**Supplementary Table 3**

| **3. Effect Sizes for Regression Models (Cohen's f^2^): Combined and Current Analysis** | | | | | |
| --- | --- | --- | --- | --- | --- |
| Cohort | Outcome | **Cohen's f^2^** | Magnitude | n1 | n2 |
|  |  |  |  |  |  |
| Current | FACIT-F | **0.665** | Large | 20 | 19 |
| Current | HAM-D | **0.225** | Medium | 20 | 20 |
| Current | Sleep Duration | **0.099** | Small | 22 | 23 |
| Combined | FACIT-F | **0.242** | Medium | 38 | 39 |
| Combined | HAM-D | **0.112** | Small | 38 | 40 |
| Combined | Sleep Duration | **0.134** | Small | 42 | 43 |

According to Cohen’s guidelines, f^2^  ≥ 0.02, f^2^  ≥ 0.15, and f^2^  ≥ 0.35 represent small, medium, and large effect sizes, respectively [S5].  *Abbreviations: FACIT-F,* Functional Assessment of Chronic Illness Therapy-Fatigue Scale*; HAM-D,* Hamilton Depression Rating Scale

**Supplementary Table 4**

| **4A. Sensitivity analysis excluding melatonin users (excluding n=5 pilot, n=6 current cohort)** | | | | | | |
| --- | --- | --- | --- | --- | --- | --- |
|  | **Current cohort (n = 17)** | | | **Combined cohort (n = 32)** | | |
|  | Baseline | Post- intervention | p-value | Baseline | Post- intervention | p-value |
| Actigraphy |  |  |  |  |  |  |
| **Sleep duration (min)** | 454.6 (81.3) | 478.2 (81.8) | 0.35 | 436.3 (87.1) | 468.4 (94.7) | **0.01** |
| **Sleep onset latency** | 10.6 (14.0) | 8.9 (15.9) | 0.40 | 11.5 (12.1) | 12.4 (16.7) | 0.64 |
| **WASO** | 60.0 (20.5) | 64.5 (42.5) | 0.49 | 50.9 (22.0) | 59.3 (41.0) | 0.91 |
| **Sleep efficiency (%)** | 83.0 (6.3) | 83.9 (9.4) | 0.44 | 83.8 (9.5) | 84.0 (8.8) | 0.52 |
| Questionnaires |  |  |  |  |  |  |
| **Fatigue (FACIT-F)** | 28.1 (12.2) | 30.7 (10.4) | **0.01** | 32.3 (12.2) | 35.2 (10.0) | **0.01** |
| **Sleep (PDSS-2)** | 17.5 (7.6) | 19.6 (6.7) | 0.83 | 16.5 (7.2) | 17.2 (6.2) | 0.81 |
| **Daytime sleepiness (ESS)** | 10.2 (6.0) | 8.5 (4.1) | 0.69 | 8.3 (5.6) | 7.4 (4.0) | 0.27 |
| **Depression (HDRS)** | 10.8 (5.1) | 9.2 (5.8) | **0.02** | 10.5 (5.3) | 9.0 (5.7) | **0.02** |
| **Anxiety (HAM-A)** | 12.5 (5.4) | 10.2 (6.9) | 0.35 | 11.7 (6.0) | 9.7 (6.3) | 0.07 |
| **Apathy (AS)** | 14.4 (9.3) | 16.2 (11.9) | 0.94 | 12.4 (8.0) | 13.0 (9.5) | 0.77 |
| Mean (SD). Results for univariate paired 1-sided t-test shown. *Abbreviations: AS,* Apathy Scale; *ESS,* Epworth Sleepiness Scale; *FACIT-F,* Functional Assessment of Chronic Illness Therapy-Fatigue Scale; *HAM-A,* Hamilton Anxiety Rating Scale; *HAM-D,* Hamilton Depression Rating Scale; *PDSS-2,* Parkinson’s Disease Sleep Scale 2; *WASO*, Wake After Sleep Onset. *Clinical cutoff scores: AS*(>14) [54], *ESS* (>10) [51], *FACIT-F*(<30) [S1], *HAM-A*(≥8) [S2], *HAM-D* (≥8) [S3], *PDSS-2*(≥15) [S4] | | | | | | |

| **4B. Sensitivity regression analysis excluding melatonin users (excluding n=5 pilot, n=6 current cohort)** | | | | | | | | | |
| --- | --- | --- | --- | --- | --- | --- | --- | --- | --- |
|  | **FACIT-F** | | | **Sleep duration** | | | **HDRS** | | |
|  | Beta | 95% CI | p-value | Beta | 95% CI | p-value | Beta | 95% CI | p-value |
| *Combined cohort* | | | |  | | |  | | |
| **Time** | 3.0 | 0.83, 5.1 | **0.007** | 31 | 8.4, 53 | **0.007** | -1.4 | -2.7, -0.11 | **0.034** |
| **Age** | 0.29 | 0.01, 0.58 | **0.043** | 2.0 | -0.36, 4.3 | 0.10 | -0.17 | -0.31, -0.02 | **0.027** |
| **Disease duration (years)** | 0.07 | -0.66, 0.79 | 0.9 | -6.2 | -12, -0.34 | **0.038** | 0.19 | -0.18, 0.57 | 0.3 |
| **Female sex** | 5.9 | -1.7, 13 | 0.13 | -14 | -76, 48 | 0.7 | 0.64 | -3.3, 4.6 | 0.7 |
| *Current cohort* | | | |  | | |  | | |
| **Time** | 3.3 | 0.79, 5.7 | **0.009** | 22 | -3.6, 48 | 0.092 | -1.9 | -3.7, 0.00 | **0.049** |
| **Age** | 0.25 | -0.20, 0.70 | 0.3 | 1.6 | -1.7, 4.9 | 0.3 | -0.19 | -0.40, 0.01 | 0.066 |
| **Disease duration (years)** | -0.11 | -1.4, 1.2 | 0.9 | -6.2 | -16, 3.2 | 0.2 | 0.37 | -0.22, 0.97 | 0.2 |
| **Female sex** | 4.4 | -7.4, 16 | 0.5 | -25 | -110, 60 | 0.6 | -1.4 | -6.7, 3.9 | 0.6 |
| Regression estimates modeling the effect of the lighting intervention effect on fatigue (*FACIT-F*, Functional Assessment of Chronic Illness Therapy-Fatigue Scale total score, Model A1 and B1), sleep duration, and depression, (*HAM-D*, Hamilton Depression Rating Scale) in the combined cohort excluding participants taking melatonin, adjusting for participant age, duration of disease, and sex. | | | | | | | | | |

**References**

[S1] Mallinson T, Cella D, Cashy J, Holzner B. Giving meaning to measure: linking Self reported

fatigue and function to performance of everyday activities. J Pain Symptom Manag 2006;31:229–41.

[S2] Matza LS, Morlock R, Sexton C, Malley K, Feltner D. Identifying HAM-A cutoffs for

mild, moderate, and severe generalized anxiety disorder. Int J Methods Psychiatr Res

2010;19:223–32.

[S3] Zimmerman M, Martinez JH, Young D, Chelminski I, Dalrymple K. Severity

classification on the Hamilton Depression Rating Scale. J Affect Disord

2013;150:384–8.

[S4] Suzuki K, Miyamoto T, Miyamoto M, Suzuki S, Numao A, Watanabe Y, et al.

Evaluation of cutoff scores for the Parkinson's disease sleep scale-2. Acta Neurol

Scand 2015;131:426–30.

[S5] Cohen J. Statistical power analysis for the behavioral sciences. 2^nd^ ed. Hillsdale (NJ): Lawrence Erlbaum Associates; 1988.
